# A Review of Surgical Outcomes and Advances for Macular Holes

**DOI:** 10.1155/2018/7389412

**Published:** 2018-04-18

**Authors:** Peng-peng Zhao, Shuang Wang, Nan Liu, Zhi-min Shu, Jin-song Zhao

**Affiliations:** ^1^Department of Ophthalmology, The Second Hospital of Jilin University, Changchun, China; ^2^Department of Ophthalmology, The Third People's Hospital of Chengdu, Chengdu, China

## Abstract

The surgical outcomes of macular holes (MHs) have improved greatly in recent years. The closure rate is as high as 90–100%, but the outcomes of some special types of MHs remain unsatisfactory. Internal limiting membrane (ILM) peeling dramatically improves the anatomic success rate, but recent studies have found that it could also cause mechanical and subclinical traumatic changes to the retina. Dyes are widely used, and apart from indocyanine green (ICG), the toxicities of other dyes require further research. Face-down posturing is necessary for MHs larger than 400 *μ*m, and the duration of this posture is determined by the type of tamponade and the case. The ellipsoid zone has been shown to be highly correlated with visual outcome and recovery. New surgical methods include the inverted ILM flap technique and the ILM abrasion technique. However, they require further research to determine their effectiveness.

## 1. Introduction

A macular hole (MH) is a full-thickness or partial-thickness defect in the macular region, and its pathogenesis can be idiopathic or result from myopia, trauma, or other causes [1, 2]. Before the application of vitrectomy, there was no specific treatment for MH [3], although some MHs were known to close spontaneously [4]. Surgery for MH has undergone great developments since Kelly and Wendel [5] first applied vitrectomy to treat MH. Both closure rate and visual recovery have improved dramatically; internal limiting membrane (ILM) peeling in particular has significantly improved the closure rate. The use of dyes and the development of microincision surgery have reduced both the duration of surgery and the risk of damage from surgery. Furthermore, a recent postoperative posturing study reported pain relief for patients, and new surgical methods may soon offer novel solutions to the remaining problems. This article reviews the outcomes of and advances in MH surgery.

## 2. The Surgical Outcomes

### 2.1. Visual Outcomes

Kelly and Wendel [5] reported that 73% patients who underwent vitrectomy resulting in successful macular reattachment experienced an improvement in visual acuity of two lines or better. With the continuous improvement in surgical techniques, the closure rate is improved and the rate of visual acuity recovery has been improved.

However, visual acuity outcomes differ by the MH type. Compared with the idiopathic MH, the postoperative visual outcomes for high myopic MH are limited. For idiopathic MHs, Tewari et al. [6] reported a final visual acuity of 20/50 following ILM peeling. Wu and Kung [4] reported that the mean logMAR visual acuity improved in a group with high myopia from 0.92 to 0.63, while a group without high myopia showed an improvement from 1.02 to 0.48. Thus, visual outcomes were less successful in highly myopic eyes. Due to the relatively less favorable outcomes of ILM peeling techniques for myopic eyes and for large and refractory MHs, the inverted flap technique was proposed in 2010. Guber et al. [7] reported that most patients with large MHs (diameter > 400 *μ*m) showed best-corrected visual acuity (BCVA) improvements of 1 to 2 lines following surgery using the inverted flap technique. Khodani et al. [8] reported that visual acuity was improved in patients with very large MHs (diameter > 1000 *μ*m), from a baseline visual acuity of 20/120 to a final visual acuity of 20/80 following surgery using the inverted flap technique.

Visual acuity improvements are known to differ depending on the stage of MH and the type of stain used in ILM peeling. Mean visual acuity has been reported to improve to 20/50 for stage 2 holes, 20/110 for stage 3 holes, and 20/145 for stage 4 holes [9]. Further, better visual outcomes following ILM peeling have been reported with brilliant blue (BB) compared to indocyanine green (ICG). Williamson and Lee [9] reported that postoperative visual acuity was 20/100 for patients who underwent surgery where ICG was used, while the postoperative visual acuity was 20/70 for patients who underwent surgery with BB.

### 2.2. Closure Rate

Before the introduction of vitrectomy to treat MH, the spontaneous closure rate for Gass stage 3 and 4 MHs was merely 4%, while that for stage 2 MHs was 11.4% [4]. Following the introduction of vitrectomy by Kelly and Wendel [5], the closure rate increased to 58%. As surgical techniques and instrumentation have improved, the closure rate has increased to as high as 90% [10].

However, as with visual outcomes, surgical outcomes differ by the MH type. Idiopathic MHs have the best outcomes, with reported closure rates ranging from 90 to 100% [6, 11–20]. Vitrectomy can reduce tangential traction at the prefoveal vitreous cortex and/or the epiretinal membranes (ERMs) as well as anteroposterior traction at the vitreoretinal interface. Compared with idiopathic MHs, the postoperative closure rates for myopic, traumatic, and large MHs are limited and these MHs require a second surgery [4, 21–29]. Traumatic MH is hypothesized to result from axial compression of the globe, which can suddenly reduce the globe's anterior-posterior diameter and cause the eyeball to expand in the equatorial direction to compensate. This change can lead to splitting of the retinal layers at the fovea [21]. The postoperative closure rate for a traumatic MH has been reported to range from approximately 83% to 92.3% [21, 30]. For a myopic MH, the closure rate has been reported to range from 63% to 90% [4, 22–25]. Although the reasons for these less favorable outcomes are not fully understood, some authors speculated that a long axial length (>30 mm) and posterior staphyloma, which can exert additional traction on the retinal surface and impede hole closure, are the two main causes for the low closure rates and limited visual recovery [4, 26].

Additionally, MH diameter is also an important issue in hole closure [28, 29, 31–35]. One study in particular indicated that when the MHs with diameters less than 400 *μ*m, the closure rate is approximately 92–97%, while MHs with diameters greater than 500 *μ*m show a closure rate of just 50% [31]. This difference in closure rates is caused by both hole diameter and the associated Gass stage. Indeed, other studies have shown that closure rate differs by stage in idiopathic MH, with lower rates being observed in stage 3 and 4 MHs compared to stage 2 MHs [9, 36–38]. Williamson and Lee [9] reported that among 351 cases, the stage 2, 3, and 4 closure rates were 95.8%, 73.0%, and 56.3%, respectively, and this difference was significant.

### 2.3. Microstructural Changes

Using histology results, postoperative MH can be divided into four types: U-shaped closures, V-shaped closures, irregular closures, and flat/open closures [39, 40]. Michalewska et al. [39] described a U-shaped closure as a normal foveal contour that results in the best visual outcomes and is present in about 45% of all patients. A V-shaped closure, described as a steep foveal contour, has been reported to be present in about 26% of cases and is associated with less favorable visual outcomes compared to U-shaped closures. Irregular closures show an irregular foveal contours and are reported to be present in about 8.8% of cases. Flat/open closures are observed in approximately 19% of cases and show foveal defects of the neurosensory retina with a flattened cuff of fluid around the hole. Visual recovery for flat/open closures is limited.

Before the advent of spectral-domain optical coherence tomography (SD-OCT), authors using time-domain OCT (TD-OCT) found that photoreceptor defects were correlated with postoperative BCVA. However, they could not determine the causing mechanisms and could only describe the foveal contour as irregular or regular due to the limited resolution of TD-OCT [41–43]. SD-OCT has a higher resolution and can enhance the intraretinal architectural morphology, especially that of the photoreceptor layer [41]. There are four distinct hyperreflective lines that can be viewed by SD-OCT: the photoreceptor inner segment/outer segment (IS/OS) junction, the external limiting membrane (ELM), the cone outer segment tips (COST), and the retinal pigment epithelium (RPE) [44]. The International Nomenclature OCT Consensus refers to the IS/OS junction as the ellipsoid zone and the COST as the interdigitation zone [45], and thus we use this nomenclature in the remainder of this review. Studies have reported that defects in the ellipsoid zone are a major reason for unsatisfactory visual recovery, and the length of the ellipsoid zone defect is negatively correlated with visual recovery. Further, as the ellipsoid zone is restored, visual acuity is expected to improve [46–51]. Other studies have found that the ELM and the interdigitation zone are also associated with visual recovery [49]. The ELM has further been shown to be correlated with the ellipsoid zone: a disrupted ELM has been reported to always be accompanied by a disrupted ellipsoid zone, but a restored or intact ELM is not always associated with an intact ellipsoid zone [50]. It has also been demonstrated that the integrity of the ELM and ellipsoid zone is the most important factor related to postoperative visual acuity [44].

A less common mechanism of MH formation is by ERM traction, usually on a lamellar macular hole (LMH) [52]. Recently, a study proposed by Pang and associates [53] found that the lamellar hole-associated epiretinal proliferation (LHEP) appears on SD-OCT images as a substantial material of homogenous medium reflectivity located on the epiretinal surface, which is a unique feature in LMH or LMH-induced full-thickness macular hole (FTMH). In addition, unlike the common configuration observed with MH, SD-OCT images show ERM-induced MHs as narrow base with wider separation in the inner retina [52].

## 3. The Surgical Techniques

### 3.1. ILM Peeling and New Surgical Methods

Several meta-analyses have indicated that ILM peeling can significantly improve initial postoperative closure rate and visual recovery and reduce the chance of a second operation [54–56]. Several studies have reported that there is no difference between peeling and nonpeeling for stage 1 and 2 MHs but there is a significant difference for stages 3 and 4 between peeling and nonpeeling, regarding the closure rate. ILM peeling releases traction caused by glial cells, which then migrate on the surface of the ILM, as it acts as a scaffold for cellular proliferation [20, 57, 58]. ILM peeling is now a standard procedure in MH surgery.

Although ILM peeling can result in improvements in MH treatment, recent research has found that it can cause mechanical and subclinical traumatic changes to the retinal nerve fiber layer (RNFL) [59, 60]. The earliest change is swelling of the arcuate retinal nerve fiber layer (SANFL) [61]. Indeed, Clark et al. [61] found that patients who underwent vitrectomy and ILM peeling presented with hypoautofluorescent arcuate striae in the macular region on infrared and autofluorescence imaging with corresponding hyperreflectantive swelling demonstrated on spectral-domain optical coherence tomography (SD-OCT). The SANFL does not appear to impact the final BCVA and can be expected to disappear in about 3 months [59]. There are two hypotheses regarding the cause of SANFL [61]. The first hypothesis is that surgical forceps cause direct damage to the retina when grasping the ILM while the second is that ILM peeling causes damage to the Müller cell endplates that are attached to the ILM.

The dissociated optic nerve fiber layer (DONFL), which is similar to the SANFL [10, 58, 59, 62], is observed as small, spindle-shaped splitting adjacent nerve fiber bundles on SD-OCT. Not all patients who undergo ILM peeling will present with the DONFL postoperatively, and there have been no significant differences observed between eyes with and those without the DONFL with respect to BCVA or macular sensitivity. The reason for DONFL presentation is also unclear, although some authors speculated that the DONFL is caused by irregularly distributed Müller cells following ILM peeling in regions that show a higher density of nerve fiber bundles in the RNFL.

Although ILM peeling is generally used for MH surgery, several surgical technique modifications have been studied in recent years. Bae et al. [20] demonstrated that the extent of ILM peeling affects the degree of postoperative metamorphopsia. They divided the radius of ILM removal into two groups of 0.75 and 1.5 disc diameter (DD) and found that a larger extent of ILM removal was related to significantly better postoperative metamorphopsia improvement. There have also been developments of new surgical methods for MHs that are associated with unsatisfactory anatomic outcomes and visual recovery, including large MHs, highly myopic MHs, and traumatic MHs. Ho et al. [63] performed the foveola nonpeeling surgery in early-stage 2 MHs. They peeled off a donut-shaped ILM, leaving a 400 *μ*m diameter ILM over the foveola. And in this way, they successfully prevented inner retinal damages, maintained the integrity of the foveolar structure, and led to better final visual acuity. In 2010, Michalewska et al. [27] first adopted the inverted ILM flap technique to treat large MHs. The authors of this study did not grasp the ILM completely but left it attached at the edges of the MH. Next, they rolled the ILM to cover the hole and left the ILM's retinal face adjacent to the vitreous cavity. This new method was shown to significantly improve the closure rates of large MHs (>400 *μ*m) and to change the flat/open closures into U-shaped or V-shaped closures. Later, the authors applied this method to highly myopic, traumatic, and other refractory MHs and were again able to achieve higher closure rates and improved visual outcomes [7, 8, 25, 28, 64–71]. However, the mechanism of the ILM flap technique is not yet clear. The results of a study by Kase et al. [70] suggest that glial cells placed on the hole may produce intermediate filaments and provoke tissue remodeling within the MH. Further, Shiode et al. [72] proposed that the ILM functions as a scaffold for the proliferation and migration of Müller cells, allowing the neurotrophic factors and basic fibroblast growth factors that are produced by the Müller cells to contribute to MH closure.

Recent studies have improved on the inverted ILM flap technique: a rolled segment of the peeled ILM into a single-layered ILM. A single-layered ILM can now be rolled and used to fill the MH [73–75]. Song et al. [73] developed a vitrectomy combined with a Viscoat- (Alcon Laboratories, Fort Worth, TX, USA) assisted single-layered inverted ILM flap technique. The use of Viscoat effectively prevents retroversion of the ILM flap during the fluid-air exchange and minimizes the toxic effect of ICG staining on the RPE. Morizane et al. [64] further developed an autologous transplantation of the ILM for refractory MHs in which the surgeon grasps the ILM flap from the ungrasped area to cover the hole for patients who undergo ILM peeling. Michalewska et al. [76] reported that the temporal inverted ILM flap technique, which involves grasping the ILM from the temporal area, reduced the incidence of the DONFL and SANFL. Chen and Yang [77] reported a technique that uses the autologous anterior or posterior lens capsule flap as a scaffold to plug the MH. Finally, Grewal and Mahmoud [78] introduced a new technique involving the use of the autologous neurosensory retinal free flap for closure of refractory myopic MHs.

Mahajan et al. [79] reported a new ILM abrasion technique for postvitrectomy in which a diamond-dusted membrane scraper is brushed over the macula in the 1 DD area surrounding the MH. This technique achieves similar results as ILM peeling (total, 94% closure) and achieves a rate of 93.5% (58/62) closure for Gass stage 3 and 4 holes [79]. They also found that this method would not penetrate the RNFL [80]. Therefore, they believe that the ILM abrasion technique is another option for MH surgery. More studies are required to determine the effectiveness of both the inverted ILM flap technique and the ILM abrasion technique.

### 3.2. Dyes and Adjuvants

Even for experienced surgeons, it is difficult to visualize the ILM during MH surgery. The application of dyes and adjuvants can make MH surgeries safer, reduce the duration of surgery, and decrease the risk of mechanical trauma to the retina.

Commonly used dyes include indocyanine green (ICG), trypan blue (TB), brilliant blue G (BBG), and acid violet 17 [58]. Adjuvants include triamcinolone acetonide (TA) and blood [58, 81].

Among these dyes, the earliest and most widely used is ICG [10], but studies have demonstrated that ICG can cause toxicity. ICG has an impact on the retinal ganglion cells (RGCs), glial cells, and RPE cells [82, 83]. Two meta-analyses have reported that eyes treated with ICG have poorer visual acuity and field outcomes than those treated with other dyes [84, 85]. Additionally, the visual field defect present after ILM removal has been shown to further progress after surgery when ICG is used [86]. TB is another widely used dye [10, 81, 87–89], but several *in vitro* experiments have shown that it causes dose- and time-dependent neurotoxicity on RGCs. However, the RGC toxicity observed for TB has been less than that observed for ICG [58, 90, 91].

Authors have also reported RPE atrophy following TB-assisted ILM peeling [92, 93]. TA has been shown to be safe and effective compared with ICG, and its usage has thus become common [81, 94–97]. The main side effect of TA is high postoperative intraocular pressure, and one *in vitro* study has reported that TA is toxic to RPE cells when applied at a normal dose [98]. However, a pig study failed to observe the same RPE atrophy 6 weeks after surgery [95]. Blood can also be used for staining [58], and one report has demonstrated that the use of whole blood before staining the ILM with BBG causes earlier and better visual postoperative rehabilitation [99]. BBG is another safe dye used in ophthalmological surgery [100]. Although ICG and TB injections have both been shown to cause retinal cell degeneration, subretinal injection of BBG had no such effect in a study using a rat model [101]. One *in vitro* experiment used ICG, TB, TA, and BBG in a rat model and reported that only ICG caused retinal cell dysfunction and structural damage [102]. Further, one electroretinogram and histopathology study used ICG, TA, and BBG in a pig eye model and reported that the cytotoxicity of ICG is significantly higher than that of TA or BBG [95]. Another experiment found that neither BBG nor TA use had a significant effect on postoperative mfERG (multifocal electroretinogram) responses or histology in pig eye [95, 103]. A meta-analysis further showed that there is no significant difference in the rate of hole closure following MH surgery using BBG versus other dyes, but significantly better recovery of postoperative visual function was present when BBG was used compared to ICG or other dyes [104]. Acid violet 17 is a dye that has been recently introduced for use in MH surgery and is specific to the ILM. This dye allows clear intraoperative visualization and provides a greater contrast than BBG. Although its safety has been confirmed at concentrations of 0.25 g/L and 0.5 g/L, further studies are required to confirm its long-term safety [58].

Indeed, further study is required to determine the toxicities of each dye, with the exception of ICG, during MH surgery.

### 3.3. Postoperative Posturing and Tamponade

When Kelly and Wendel [5] first introduced vitrectomy for MH treatment, they used room air to fill the vitreous cavity and required that the patients stay face-down for one week. Face-down posturing has thus become a standard of MH treatment.

However, face-down posturing is accompanied by great inconvenience, and some patients, especially children and the elderly, cannot tolerate it. Therefore, there is still debate about the necessity and duration of face-down posturing [105, 106]. A meta-analysis by Hu and colleagues [107] found that patients who stay face-down show better anatomic outcomes than those who do not. However, when the MH diameter was <400 *μ*m, face-down posturing showed no significant difference in anatomic success, while face-down posturing was associated with a higher success rate when the MH diameter was >400 *μ*m [108]. Thus, face-down posturing currently seems to be necessary, especially for large MHs.

The duration of face-down posturing is strongly related to the type of tamponade that is used in MH surgery. Two kinds of tamponades can be used in the vitreous cavity: gas and silicone oil. Gas plays a very important role in MH surgery because air not only can provide scaffolds for cellular proliferation but also can cause the extrusion of subretinal fluid from surface tension [109]. Although room air was originally used for MH surgery, long-lasting gas can also be applied [105]. Then, long-lasting gas became widely used to improve the effect of the gas, and the duration was extended to 1 month. Recently, some researchers have found that room air can have similar outcomes to a long-lasting gas, but the hole diameters in their cases were small, so the conclusions that can be drawn are limited [110, 111]. Commonly used long-lasting gases include sulfur hexafluoride (SF_6_) and perfluoropropane (C_3_F_8_) [112], and no significant differences in anatomic success or visual outcomes have been reported between these [108, 112–114]. When long-lasting gas became widely used as an improved tamponade, the duration was extended to 1 month. Recently, two studies have reported that room air can have similar outcomes to long-lasting gas, but the conclusions that can be drawn from these studies are limited because they only included holes with small diameters [110, 111]. The degree of gas fill also affects MH closure, as a gas fill above at least 65% on postoperative day 4 has been shown to reduce the risk of poor gas-macula contact and surgical failure [106]. Kannan et al. [115] reported that a smaller volume of SF_6_ can be used for a longer duration in order to achieve good surgical outcomes at a decreased cost.

Silicone oil, which is also used as a tamponade, is available in heavy and light varieties [116]. There is no consensus about which of these is better with respect to closure rate [117], but one report found that high intraocular pressure is more common following the use of heavy silicone oil [118]. Heavy silicone oil can also cause complications such as intraocular inflammation reaction, media opacification, and secondary glaucoma [119, 120]. Face-down posturing is not strictly necessary when using silicone oil [83, 116], and heavy silicone oil only requires that the patient be lied flat [116]. Silicone oil is mainly used for patients who cannot tolerate face-down posturing (e.g., children) [121], for large MHs [21], for MHs that remain open after the first operation [122], for highly myopic MHs with retinal detachment, and for cases involving posterior staphyloma [117]. Studies have demonstrated that initial closure rates and visual outcomes are lower with silicone oil than with gas [111, 123–126], mainly because of silicone oil's lower buoyant force [124], toxic effect on photoreceptor cells, and the cases that are chosen for silicone oil use [21, 83, 124]. In addition, the use of silicone oil requires another operation to remove it. Finally, a recent study has indicated that the use of ILM flap repositioning and autologous gluconated blood clumps as a macular plug is effective in achieving satisfactory hole closure with statically significant functional improvements and in reducing the occurrence of cataract and high intraocular pressure after surgery [127].

Apart from the tamponade type, the duration of face-down posturing is also dependent on the MH itself. One study indicated that most MHs close during the first postoperative day [128]. Research using short-lasting gases and shorter durations has shown similar outcomes [83, 109, 129–131]. Tatham and Banerjee's meta-analysis found that a duration of 24 h versus 5–10 days resulted in no significant differences in the closure rate [132]. Iezzi and Kapoor [133] reported that MH surgery using broad ILM peeling, 20% SF_6_ gas, and no face-down positioning is highly effective for idiopathic MH. More studies are needed to investigate short durations of face-down posturing.

## 4. Conclusion

There is no doubt that MH surgery has made huge progress leading to better accuracy and convenience and less damage. The continual development of new instruments helps surgeons to better assess microstructural changes, while new surgical methods provide a promising direction for treatment. However, these new advances are still being explored, and more research is needed in order to develop more definitive conclusions.
